# The Deterioration of Agronomical Traits of the Continuous Cropping of Stevia Is Associated With the Dynamics of Soil Bacterial Community

**DOI:** 10.3389/fmicb.2022.917000

**Published:** 2022-06-16

**Authors:** Xinjuan Xu, Qingyun Luo, Qichao Wei, Shangtao Jiang, Caixia Dong, Mohammad Omar Faruque, Zhongwen Huang, Zhenghua Xu, Changxi Yin, Zaibiao Zhu, Xuebo Hu

**Affiliations:** ^1^Institute for Medicinal Plants, College of Plant Science and Technology, Huazhong Agricultural University, Wuhan, China; ^2^College of Life Science and Technology, Collaborative Innovation Center of Modern Biological Breeding, Henan Institute of Science and Technology, Xinxiang, China; ^3^National-Regional Joint Engineering Research Center in Hubei for Medicinal Plant Breeding and Cultivation, Huazhong Agricultural University, Wuhan, China; ^4^Medicinal Plant Engineering Research Center of Hubei Province, Huazhong Agricultural University, Wuhan, China; ^5^Innovation Academy of International Traditional Chinese Medicinal Materials, Huazhong Agricultural University, Wuhan, China; ^6^Department of Traditional Chinese Medicinal Materials, College of Horticulture, Nanjing Agricultural University, Nanjing, China; ^7^Jiangsu Provincial Key Lab of Solid Organic Waste Utilization/Educational Ministry Engineering Center of Resource-Saving Fertilizers/Jiangsu Collaborative Innovation Center of Solid Organic Waste, Nanjing Agricultural University, Nanjing, China; ^8^Ethnobotany and Pharmacognosy Lab, Department of Botany, University of Chittagong, Chittagong, Bangladesh

**Keywords:** soil physicochemical properties, bacterial community, continuous cropping, rhizosphere soil, stevia

## Abstract

*Stevia rebaudiana* Bertoni is grown worldwide as an important, natural sweetener resource plant. The yield of steviol glycosides (SVglys) is greatly influenced by continuous cropping. In this study, we collected the roots, rhizosphere soils, and bulk soils from 2 years of continuous cropping (Y2) and 8 years of continuous cropping (Y8). A high-throughput sequencing technology based on Illumina Hiseq 2500 platform was used to study the structure and diversity of bacterial communities in the roots and soils of stevia with different years of continuous cropping. The results demonstrated that although the content of a group of SVglys was significantly increased in stevia of long-term continuous cropping, it inhibited the growth of plants and lowered the leaf dry weight; as a result, the total amount of SVglys was significantly decreased. Meanwhile, continuous cropping changed the physicochemical properties and the bacterial composition communities of soil. The different sampling sources of the root, rhizosphere soil, and bulk soil had no impact on the richness of bacterial communities, while it exhibited obvious effects on the diversity of bacterial communities. Continuous cropping had a stronger effect on the bacterial community composition in rhizosphere soil than in root and bulk soil. Based on linear discriminant analysis effect size (LEfSe), in the rhizosphere soil of Y8, the relative abundance of some beneficial bacterial genera of *Sphingomonas, Devosia, Streptomyces*, and *Flavobacterium* decreased significantly, while the relative abundance of *Polycyclovorans, Haliangium*, and *Nitrospira* greatly increased. Moreover, the soil pH and nutrient content, especially the soil organic matter, were correlated with the relative abundance of predominant bacteria at the genus level. This study provides a theoretical basis for uncovering the mechanism of obstacles in continuous stevia cropping and provides guidance for the sustainable development of stevia.

## Introduction

Stevia [*Stevia rebaudiana* (Bertoni) Hemsl.] is a perennial medicinal herb of the family *Asteraceae*. Steviol glycosides (SVglys) have many advantages including high concentration (~4–20% of leaf dry weight), high sweetness (~300–450 times of sucrose), and low heat (close to zero calories) (Karimi et al., 2019). Belonging to a kind of terpenoid mixture, SVglys mainly consist of Stevioside (STV) and Rebaudioside series; among them, STV and Rebaudioside A (RA) are higher than other ingredients (Tavarini et al., 2015). SVglys have the characteristics of easy dissolution, heat resistance, stability, harmfulness, and certain adjuvant therapeutic effects on diabetes, obesity, and dental caries (Díaz-Gutiérrez et al., 2020). With the widespread usage of SVglys in food, beverage, and other industries as a natural sweetener, stevia is widely cultivated in the world as a new source of sugar. The continuous cropping inevitably occurs because the plant is normally grown around the processing factories for SVgly extraction. However, continuous cropping usually leads to poor crop growth, low yield, poor quality, and susceptibility to diseases (Yang et al., 2011; Wacal et al., 2019; Liu et al., 2020), which seriously restricts the sustainable cropping. Soil factors are considered to be the main causes of continuous cropping obstacles, such as nutrient imbalance, property deterioration, and microbial community structure change (Wang et al., 2020; Li et al., 2021).

Soil microorganisms and plant roots interact with each other mainly in the rhizosphere (Qiao et al., 2017). Plant rhizosphere refers to the soil around the root system that changes physicochemical and biological characteristics due to the growth and activity of plant roots (Berendsen et al., 2012). This is a highly diversified microbial habitat, in which, plants rely on the exchange between roots and rhizosphere microorganisms to obtain nutrients, promote growth, and inhibit diseases (Wu et al., 2015; Garcia et al., 2016). Rhizosphere bacteria with the most abundant are beneficial to the soil to remain in a healthy and productive state (Li et al., 2019). Apart from soil fertility, continuous cropping also changes the quantity and activity of rhizosphere microorganisms (Castrillo et al., 2017; Wang et al., 2018). For example, the relative abundances of the *Firmicutes, Actinobacteria, Bacteroidetes*, and *Basidiomycota* phyla were depleted during years of vanilla monoculture (Xiong et al., 2015). Similarly, after continuous cropping of *Radix pseudostellariae, Pseudomonas* spp., *Burkburkella* spp., and *Bacillus pumilus* in the rhizosphere decreased significantly (Wu et al., 2019), while the *Fusarium* genus, one major pathogen of plant root rot, showed increasing tendency with continuous cropping of American ginseng (*Panax quinquefolium* L.) (Fan et al., 2021). These observations indicated that continuous cropping destroyed the balance of original microbial flora and resulted in the imbalance of rhizosphere microecology (Dong et al., 2016; Li et al., 2021), which would have an important impact on the quality of medicinal plants. Zhu et al. (2021) found that different soil microenvironments significantly affected the growth and active components of *Angelica sinensis*, and the key bacterial species may be the key factor determining the quality of *A. sinensis*, which indicated that the soil microenvironment contributed to the growth and accumulation of active components of medicinal plants.

High-throughput sequencing provides a new strategy for the study of rhizosphere microbial communities (Xia et al., 2019). By performing large-scale soil microbial sequencing, the composition and distribution of bacteria colonized in the plant's roots and soil can be explored. Moreover, the changes in bacterial community components at different taxonomy levels can be traced (Wang et al., 2021). So far, an in-depth understanding of soil bacterial community diversity in stevia, especially for the situation of continuous cropping, is yet to be explored. Therefore, based on 16s rDNA high-throughput sequencing technology, this study aimed to investigate the differences in bacterial community composition and the structure in the rhizosphere of stevia after short-term and long-term continuous cropping, as well as to analyze the possible mechanisms and the impacts on rhizosphere of ecological processes. This study also tried to explore the relationship between the bacterial community and soil environmental factors, which provided valuable clues for bioremediation of continuous cropping obstacles of stevia.

## Materials and Methods

### Site Description and Experimental Design

The study area was located in Guardian Town, Mingguang City, Anhui Province, China (32°77'N, 117°99'E) with an altitude of 30 m above sea level. The climate belongs to the transition zone between subtropical and temperate zones. The average annual temperature is 15°C, the annual rainfall is 940 mm, the annual sunshine is 2,210 h, and the frost-free period is 219 days. The test site was a continuous cropping field of stevia, where no other crops were planted before or after stevia harvest each year. This experiment was set for 2 years of continuous cropping (Y2) and 8 years of continuous cropping (Y8), which were performed in sandy loam. The physicochemical properties of Y2 were tested with pH 8.21, total nitrogen (TN) 1.19 g/kg, organic matter (OM) 14.86 g/kg, available phosphorus (AP) 93.41 mg/kg, and available potassium (AK) 72.00 mg/kg; while for the Y8, the corresponding profiles were pH 7.57, TN 1.10 g/kg, OM 13.25 g/kg, AP 72.61 mg/kg, and AK 80.33 mg/kg. Before transplanting, nitrogen, phosphorus, and potassium compound fertilizer (N:P_2_O_5_:K_2_O = 15:15:15, 750 kg/ha) and rotten chicken manure were applied as base fertilizer, and 300 kg/ha compound fertilizer was applied after transplanting.

### Sampling and Measurements

#### Collection of Soil and Plant Samples

The samples were collected before the flowering stage of stevia, and 18 plants were randomly selected for the sampling method (“S” shape) for 6 replicates (3 plants for each replicate). During the sampling, the root and the soil around the root were all excavated with a spade and immediately put into an icebox, which was transported to the laboratory for subsequent experiments. The soil shaken off was used as bulk soil (BS), and the soil adhering to the root surface brushed off with a brush was used as rhizosphere soil (RS). The collected soil samples were divided into two parts, one part is for the determination of physicochemical indexes after natural air drying, and the other part is that soil and root samples cleaned with sterile water were placed in a −80°C refrigerator for DNA extraction. The microbial test samples were named root (Y2.R and Y8.R), rhizosphere soil (Y2.RS and Y8.RS), and bulk soil (Y2.BS and Y8.BS), respectively. At the same time, the leaves of the above-ground plants were taken and dried naturally for SVglys determination.

#### Analysis of Soil Chemical Properties, Plant Growth, and SVgly Content

The soil samples were sieved through a 2-mm mesh prior to chemical analysis. Soil pH was determined using a pH electrode in soil–water (1:2.5 W/V) after shaking for 30 min. OM, TN, AP, and AK of soil were determined using a previously described method (Shen et al., 2015). Plant height, stem diameter, and leaf dry weight were measured using a meter ruler, vernier caliper, and electronic balance, respectively. The soil and impurities attached to the root system were removed and rinsed with clean water. Root pictures were obtained after scanning using a scanner (Epson V700), and indicators of root length, root surface area, root diameter, and root volume were obtained using the WinRHIZO root analysis system (Pro2007d). The leaf sample was ground thoroughly in a mortar. After passing through a 60-mesh sieve, accurately weighed 50 g of the sample, added 1.5 ml of 50% ethanol, ultrasonically extracted for 40 min, centrifuged at 10,000 r/min for 10 min, the supernatant was filtered by 0.45 μm organic microporous membrane, and the contents of STV, RA, Rebaudioside C (RC), Rebaudioside D (RD), Rebaudioside M (RM), and Rebaudioside F (RF) were detected according to Liu et al. (2010).

#### DNA Extraction, Amplification, and Sequencing

The total DNA was extracted from root and soil samples using the Minkagene Plant DNA Kit and Advanced Soil DNA Kit, respectively. NanoDrop was used to detect DNA concentration and purity. The amplification primers were 338F (5'-ACTCCTACGGGAGGCAGCA-3') and 806R (5'-GGACTACHVGGGTWTCTAAT-3'), and the 16S rDNA V3-V4 region sequences were amplified. The polymerase chain reaction (PCR) amplification system was as follows: 25 μl 2X Premix Taq, 1 μl Primer-F (10 mM), 1 μl Primer-R (10 mM), 3 μl DNA (20 ng/μl), and 20 μl Nuclease-free water. PCR reaction parameters were as follows: 94°C denaturation for 5 min, 94°C for 30 s, 52°C for 30 s, 72°C for 30 s, a total of 30 cycles, and extension at 72°C for 10 min. The concentrations of PCR products were compared using the GeneTools analysis software (version 4.03.05.0, SynGene). The required volumes of each sample were calculated according to the principle of equal mass, and the PCR products were mixed. Reagent for gel recovery using E.Z.N.A.^®^ Gel Extraction Kit and target DNA fragments were recovered using TE buffer elution. The library was built according to the NEBNext^®^ Ultra TM DNA Library Prep Kit for the Illumine^®^ standard process. The library was sequenced on an IlluminaHiseq2500, and 250 bp paired-end reads were generated (Guangdong Magigene Biotechnology Co., Ltd., Guangzhou, China).

### Bioinformatics and Statistical Analyses

The raw sequenced data were analyzed according to the Qiime2 platform (version 2020.11.0) (Bolyen et al., 2019). After optimizing statistics and removing impurities, the original sequencing data were flatted, and then the effective sequences were clustered into the operational taxonomic unit (OTU) according to 97% similarity using Usearch (version 10.0.240; http://www.drive5.com/usearch/) (Edgar, 2010) with default parameters. The taxonomic assignment for each OTU was carried out using Usearch - sintax with a cutoff of 0.8. The search database for the assignment was the SILVA 16S rRNA database (Quast et al., 2013). The taxonomic assignment results were shown in seven levels (i.e., Kingdom, Phylum, Class, Order, Family, Genus, and Species). Alpha diversity (Chao richness and Shannon's diversity) statistics for each sequencing sample was performed using Usearch alpha_div (version 10.0.240). Statistical analysis of the sequencing data was conducted in the R environment (version 3.5.1). Beta diversity (principal coordinate analysis, PCoA) based on Bray Curtis dissimilarities was performed using R package “vegan”. We performed LEfSe analysis to discover the significant differences in microbiota taxa between the long- and short-term groups. In this study, LEfSe analysis was carried out using microbiota taxa in phylum and genus level, and taxa were identified with statistically significant (*p* < 0.05) and linear discriminant analysis (LDA) score > 3. The canonical correspondence analysis (CCA) was used to evaluate the effects of soil physicochemical properties on microbial community structure. The SAS 9.12 was used for the analysis of variance and correlation, and the least significance difference (LSD) method was used for multiple comparisons with the significance level set to *p* < 0.05. The graphs were created using GrapPad Prism 7.

## Results

### Effect of Continuous Cropping on Plant Growth, SVgly Content, and Soil Physicochemical Properties

**Figure 1:**
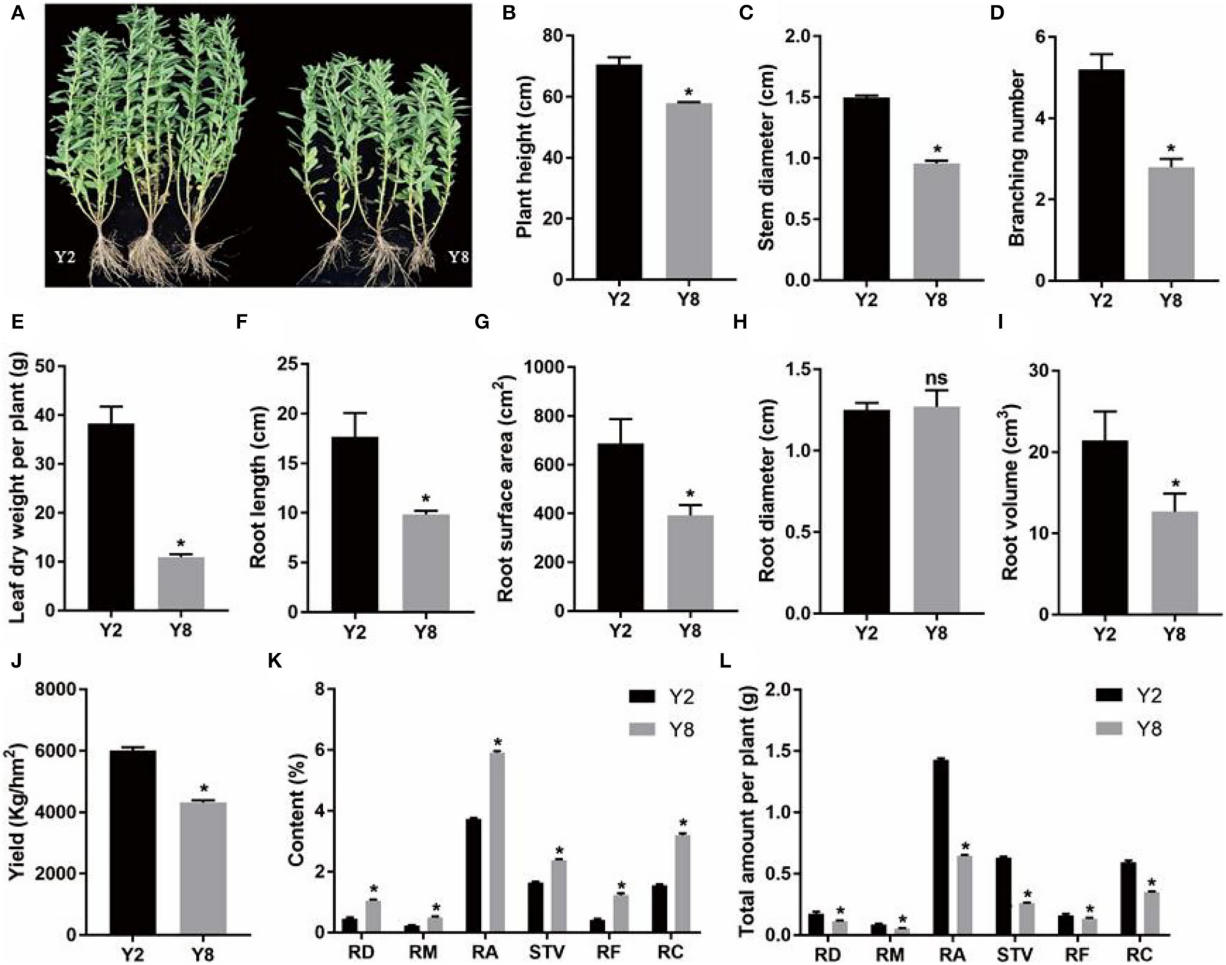
The characteristics and comparison of plant growth and the content of steviol glycoside (SVgly) content between 2 years of continuous cropping (Y2) and 8 years of continuous cropping (Y8). **(A)** Stevia plants grow after Y2 and Y8, respectively. **(B)** Plant height. **(C)** Stem diameter. **(D)** Branching number. **(E)** Leaf dry weight per plant. **(F)** Root length. **(G)** Root surface area. **(H)** Root diameter. **(I)** Root volume. **(J)** Yield. **(K)** The content of SVglys. **(L)** The total amount of SVglys per plant. The total amount of SVglys per plant was based on results of dry leaf weight per plant and the content of SVglys. RD, RM, RA, RF, RC, and STV represent Rebaudioside D, Rebaudioside M, Rebaudioside A, Rebaudioside F, Rebaudioside C, Stevioside, respectively. ns means nonsignificant. *indicates significant differences between Y2 and Y8 (*p* < 0.05).

**Figure 2:**
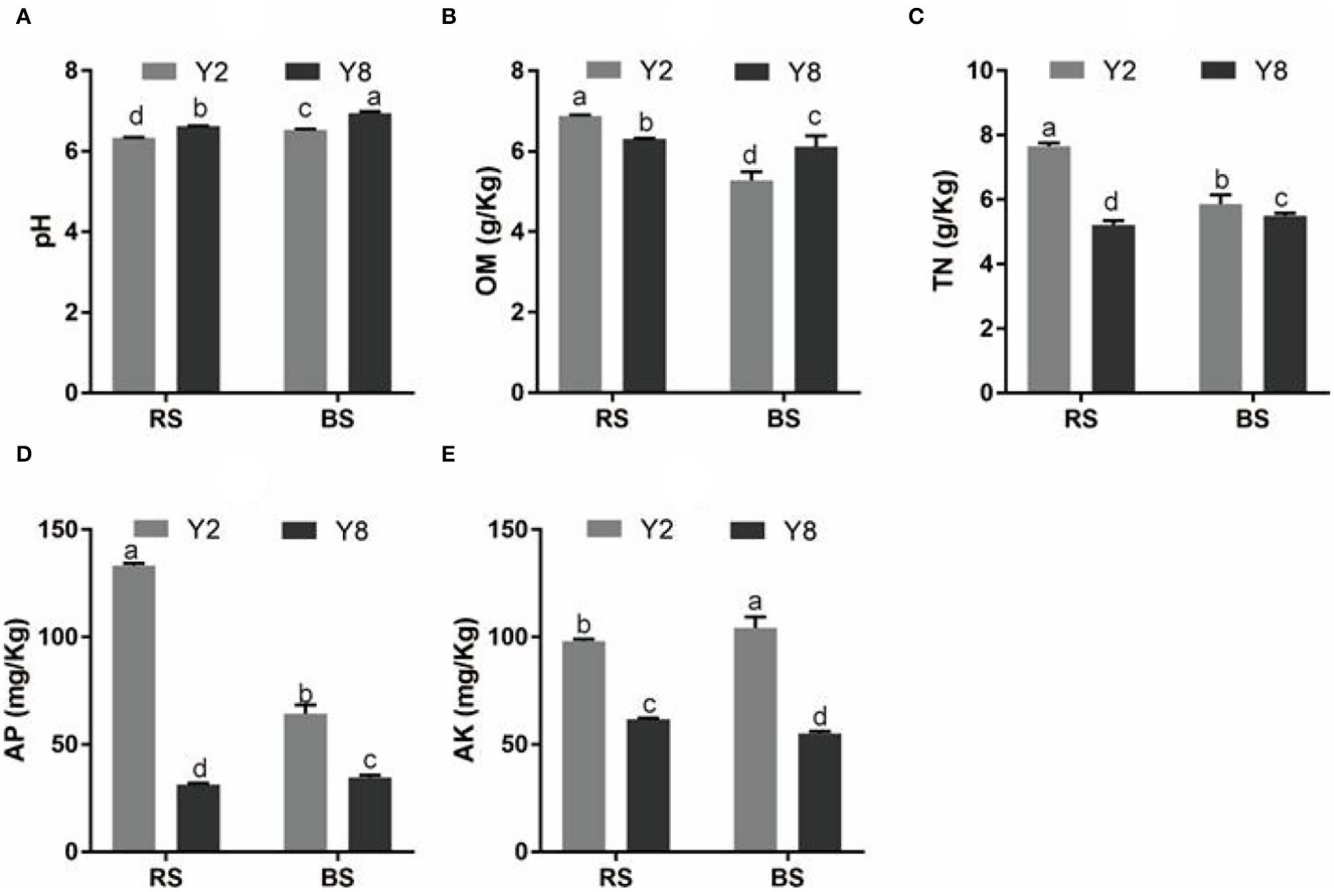
A comparison of soil physicochemical properties of rhizosphere and bulk soil of continuous cropping of stevia for 2 and 8 years. **(A)** pH. **(B)** OM. **(C)** TN. **(D)** AP. **(E)** AK. pH, pH value; OM, organic matter; TN, total nitrogen; AP, available phosphorus; AK, available potassium. Y2, 2 years of continuous cropping; Y8, 8 years of continuous cropping; Y2.RS, rhizosphere soil of 2 years of continuous cropping; Y8.RS, rhizosphere soil of 8 years of continuous cropping; Y2.BS, bulk soil of 2 years of continuous cropping; Y8.BS, bulk soil of 8 years of continuous cropping. The different lowercase letters in the same column indicate significant differences between different treatments (*p* < 0.05).

The continuous cropping severely inhibited the growth of stevia (Figure 1A). Except for root diameter, other plant growth parameters, namely, plant height, stem diameter, branching number, leaf dry weight per plant, root length, root surface area, root diameter, root volume, and yield of Y8 were significantly lower (*p* < 0.05) than that of Y2 (Figures 1B–J). Although the content of SVglys in Y8 was higher than in Y2 (Figure 1K), the lower biomass still determined its lower total amount (Figure 1L). In addition, the soil TN, AP, and AK contents significantly decreased, whereas soil pH significantly increased (*p* < 0.05) after 8 years of continuous cultivation (Figure 2).

### Impacts of Continuous Cropping on Bacterial Community Composition in Plant's Root and Soil

**Figure 3:**
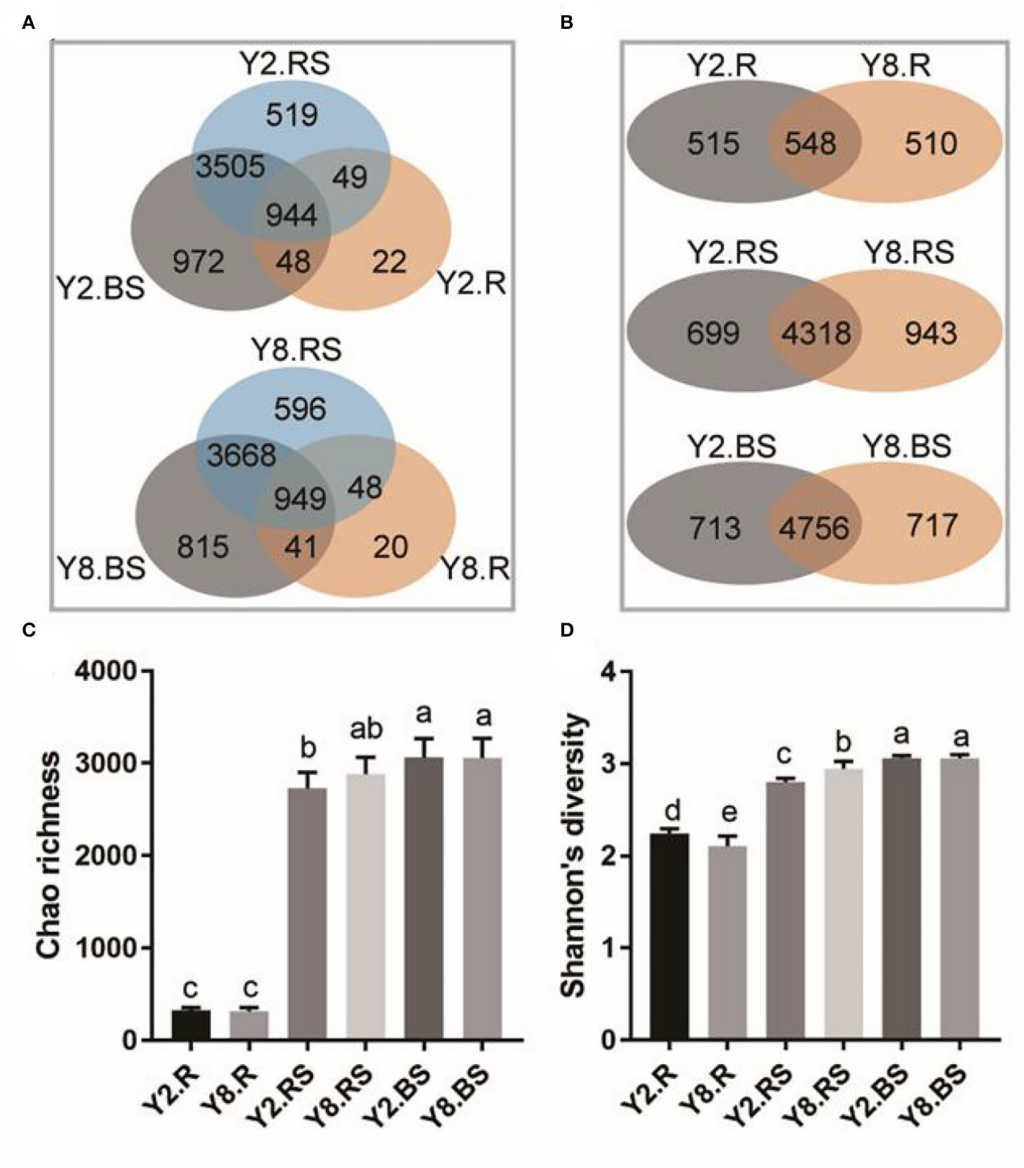
The distribution of operational taxonomic units (OTUs) and the bacterial community richness and diversity among different comparison groups. **(A)** Venn diagram of different parts of Y2 and Y8. **(B)** Venn diagram of R, RS, and BS between Y2 and Y8. **(C)** Chao richness. **(D)** Shannon's diversity. Y2.R, root of 2 years of continuous cropping; Y8.R, root of 8 years of continuous cropping; Y2.RS, rhizosphere soil of 2 years of continuous cropping; Y8.RS, rhizosphere soil of 8 years of continuous cropping; Y2.BS, bulk soil of 2 years of continuous cropping; Y8.BS, bulk soil of 8 years of continuous cropping. The different lowercase letters indicate significant differences between different treatments (*p* < 0.05).

In this study, there were 6,443 distinct OTUs observed among all subsamples, which were from an average of 460,261 sequences across each subsample. The results showed that the number of OTUs in the root was lower than that in rhizosphere soil and bulk soil (Figure 3A). The overlap number of OTUs in the Venn diagram reflected the correlation degree of bacterial diversity. The total number of OTUs in Y2 was 6,059, and the specific OTUs in the root, rhizosphere soil, and bulk soil accounted for 0.36, 8.57, and 16.04%, respectively. The total number of OTUs in Y8 was 6,137, of which the specific OTUs in the root, rhizosphere soil, and bulk soil accounted for 0.33, 9.71, and 13.28%, respectively. In addition, the OTU numbers that were commonly detected among root, rhizosphere soil, and bulk soil accounted for 15.58 and 15.46% in Y2 and Y8, respectively. Overall, the number of bacterial species detected in roots was much lower than that in soil samples, and they diverged across different years of continuous cropping (Figure 3B). The Chao richness and Shannon's diversity were calculated to further investigate the community richness and diversity across each sample. The microbial richness in roots was lower than that in soil samples separately in both Y2 and Y8. Across different continuous cropping years, the richness has no significant differences in the root, rhizosphere soil, and bulk soil (Figure 3C). The microbial richness varied along with the continuous cropping but without significance, while the species diversity values were significantly different in the root and rhizosphere soil (Figure 3D).

**Figure 4:**
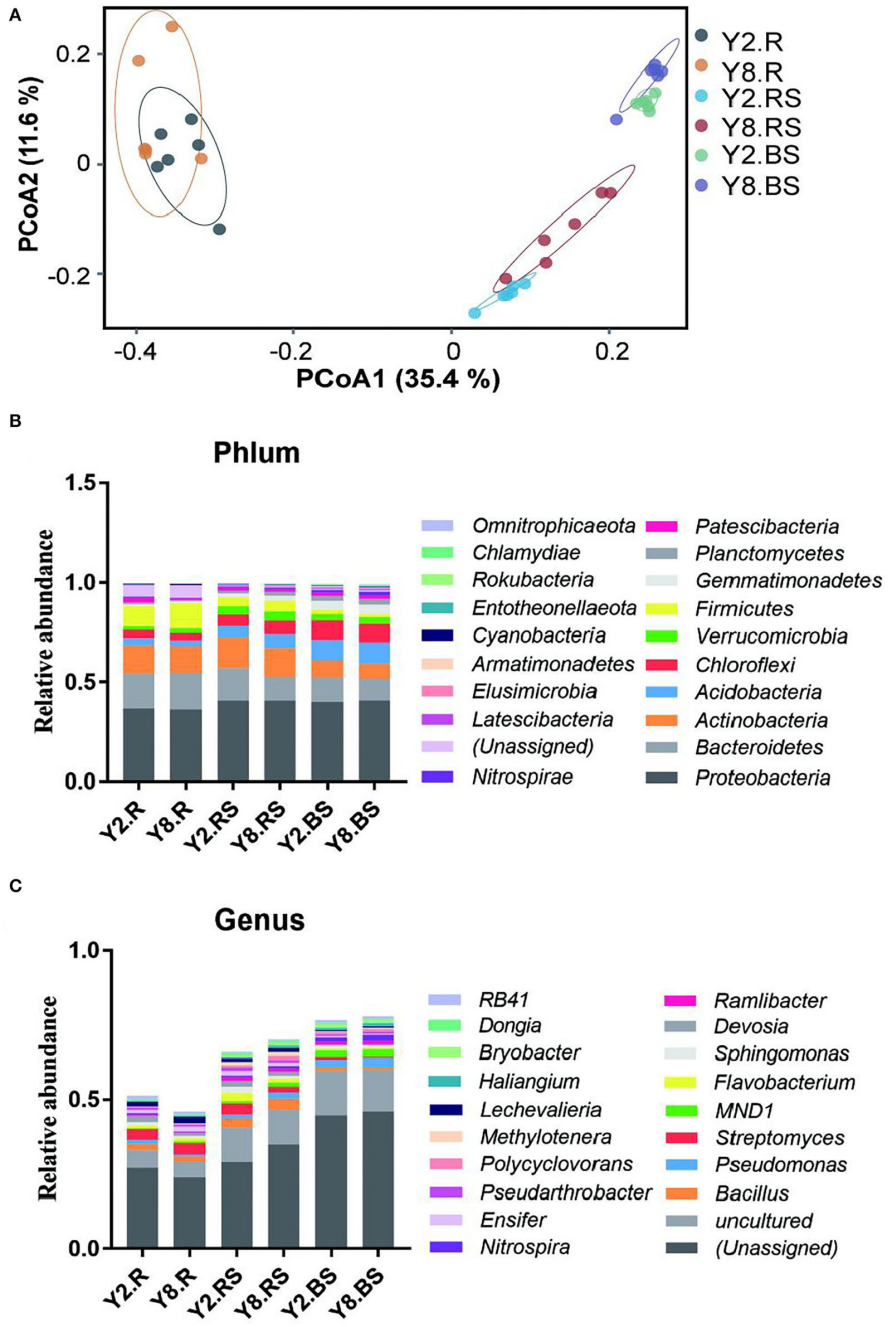
The microbiome compositions and abundance distribution of bacterial taxa in all the groups. **(A)** Principal coordinate analysis (PCoA) of the microbial community among all the groups based on the Bray–Curtis distance. **(B)** The composition of the bacterial community at the phylum level. **(C)** The composition of the bacterial community at the genus level. Y2.R, root of 2 years of continuous cropping; Y8.R, root of 8 years of continuous cropping; Y2.RS, rhizosphere soil of 2 years of continuous cropping; Y8.RS, rhizosphere soil of 8 years of continuous cropping; Y2.BS, bulk soil of 2 years of continuous cropping; Y8.BS, bulk soil of 8 years of continuous cropping.

The PCoA was performed to compare the similarity or dissimilarity of bacterial communities among different samples (Figure 4A). The two main coordinates explained 47.00% of the microbial community changes among all the samples, of which PC1 explained 35.4% of the variation and PC2 explained 11.6% of the variation. Meanwhile, PCoA showed that all the samples were grouped into three clusters. The root samples, rhizosphere soil samples, and bulk soil samples between Y2 and Y8 were much closer due to their similar bacterial community structure. The root samples differed mostly from soil samples, and the two root samples were not farther apart. This indicated that soil bacterial community structures were distinctly different from the root samples, and it suggested that the structure of the soil bacterial community was affected by continuous stevia cropping. To illustrate the above results, we further investigated the abundance divergence of microbiome compositions among all the six groups in six taxonomy classification levels, and only the rhizosphere soil showed significant differences between Y2 and Y8 (Supplementary Table 1).

The abundance distribution of bacterial composition in each group at the phylum and genus levels is shown in Figures 4B,C. At the phylum level, the top 10 bacterial phyla (>0.1%) in Y2 and Y8 were *Proteobacteria, Bacteroidetes, Actinobacteria, Actinobacteria, Chloroflexi, Verrucomicrobia, Firmicutes, Gemmatimonadetes, Planctomycetes*, and *Patescibacteria*. It was noteworthy that all the phyla showed significant differences among the six treatments except for *Proteobacteria* (*p* < 0.05). The relative abundance of *Actinobacteria, Chloroflexi, Gemmatimonadetes*, and *Planctomycetes* in bulk soils was significantly higher than in roots and rhizosphere soils, while *Actinobacteria* exhibited the opposite trend. In addition, only *Actinobacteria, Gemmatimonadetes*, and *Planctomycetes* in rhizosphere soils showed significant differences between Y2 and Y8, the same as *Patescibacteria* in roots. At the genus level, *Bacillus, Pseudomonas, Streptomyces, MND1, Flavobacterium, Sphingomonas, Devosia, Ramlibacter, Nitrospira*, and *Ensifer* were the top 10 genera, followed by *Pseudarthrobacter, Polycyclovorans, Methylotenera, Lechevalieria, Haliangium, Bryobacter*, and *Dongia*.

### Identification of Dominant Bacteria That Are Related to Continuous Cropping

**Figure 5:**
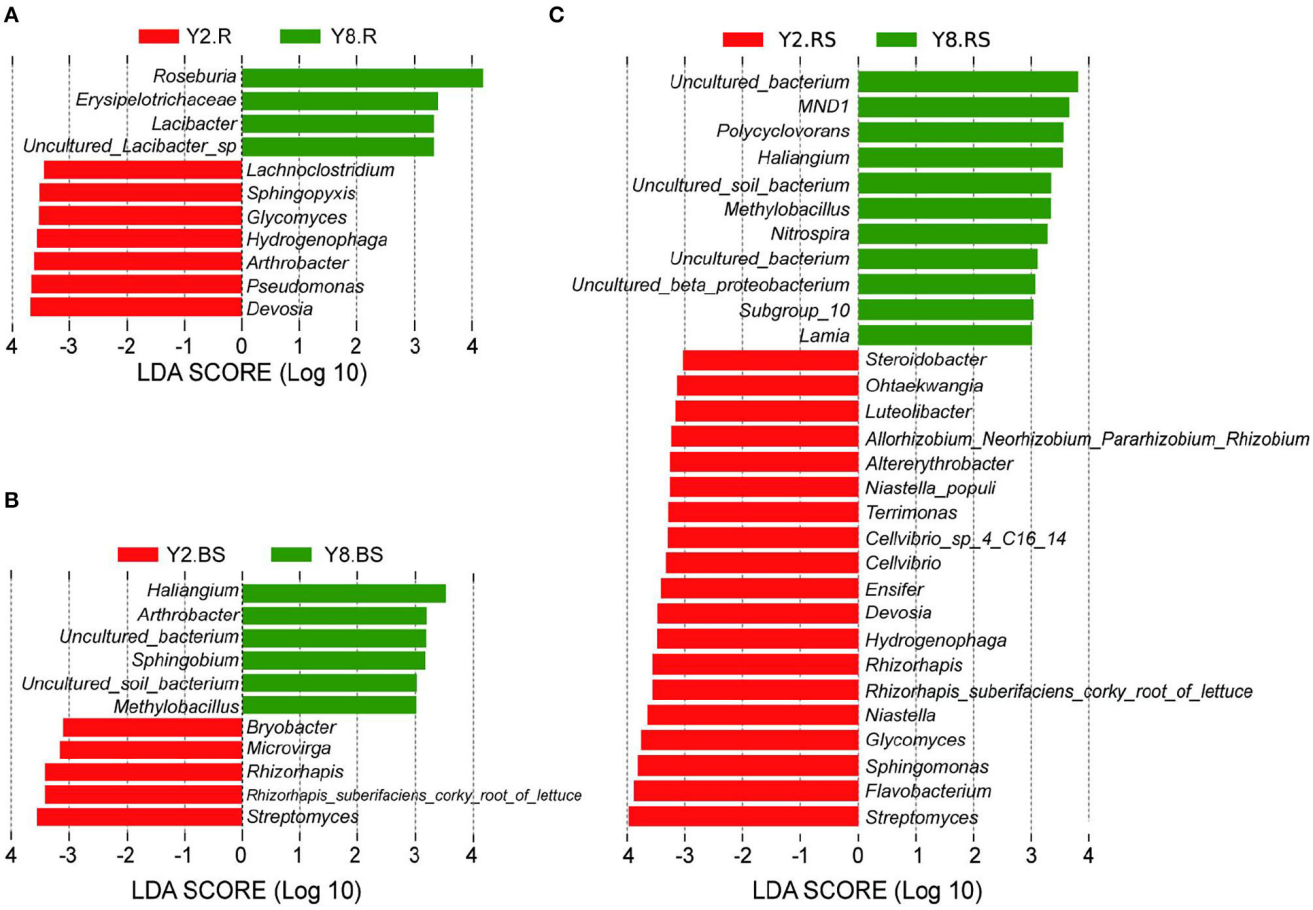
The enrichment analysis of bacterial taxa in all the groups at the genus level [linear discriminant analysis (LDA) > 3]. **(A)** Linear discriminant analysis effect size (LEfSe) analysis with significant differences based on Y2.R and Y8.R. **(B)** LEfSe analysis with significant differences based on Y2.BS and Y8.BS. **(C)** LEfSe analysis with significant differences based on Y2.RS and Y8.BS. Abbreviations: Y2.R, root of 2 years of continuous cropping; Y8.R, root of 8 years of continuous cropping; Y2.RS, rhizosphere soil of 2 years of continuous cropping; Y8.RS, rhizosphere soil of 8 years of continuous cropping; Y2.BS, bulk soil of 2 years of continuous cropping; Y8.BS, bulk soil of 8 years of continuous cropping.

To gain more insights into the variation in microbial abundance and composition during the continuous cropping, we narrowed our focus to detect differences at the genus level between Y2 and Y8. The LEfSe identified differently abundant taxa between groups and estimated each significantly different taxon's effect size. The LEfSe was performed to identify a specific genus and to search for a statistically significant biomarker at the genus level. The results (Figure 5) showed 11, 30, and 11 bacterial taxa with significant differences in root samples, rhizosphere soils, and bulk soils, respectively. For the Y2 group, *Devosia* in roots, *Streptomyces* in bulk soils, and *Streptomyces, Flavobacterium, Sphingomonas, Glycomyces*, and *Niastella* in rhizosphere soils were the most abundant types of genera, respectively, whereas for the Y8 group, the most abundant ones were *Roseburia* in the root, *Haliangium* in bulk soils, and *Polycyclovorans, Haliangium, Methylobacillus, Nitrospira*, and *Lamia* in rhizosphere soils, respectively.

### Correlation Analysis Between Soil Microbiome Composition and Soil Environmental Variables

**Figure 6:**
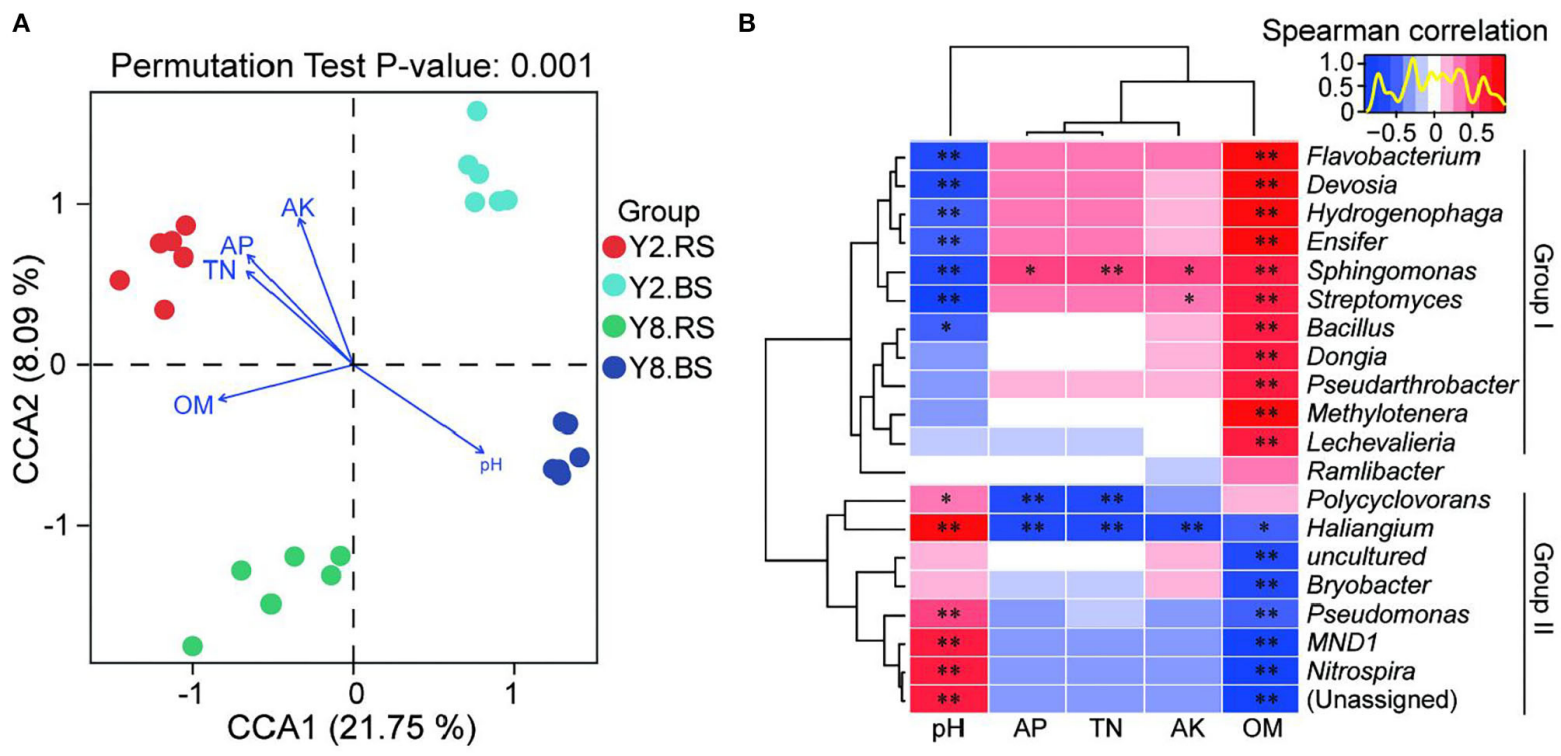
Correlation analysis between soil microbiome compositions and soil environmental variables. **(A)** Canonical correspondence analysis (CCA) based on the OTU level of soil bacteria and soil environmental variables. **(B)** Heatmap of Spearman's correlation coefficients combined with a cluster analysis between the top 20 bacterial genus and physicochemical characteristics of rhizosphere soil and bulk soil. The red color represents a positive relationship, the blue color represents negative relationships, and the darker color indicates higher correlation. Y2.RS, rhizosphere soil of 2 years of continuous cropping; Y8.RS, rhizosphere soil of 8 years of continuous cropping; Y2.BS, bulk soil of 2 years continuous of cropping; Y8.BS, bulk soil of 8 years of continuous cropping. pH, soil pH; OM, organic matter; TN, total nitrogen; AP, soil available phosphors; AK, soil available potassium. *p*-value is correlation test result (*p < 0.05; **p < 0.01).

Canonical correspondence analysis (CCA) at the OTU level reflected the relationship between physicochemical properties and the bacterial community of soil (Figure 6A). The four groups were completely separated, and all the soil physicochemical properties significantly affected the bacterial community structure, and the correlation coefficients were 0.929 (pH), 0.922 (AK), 0.881 (AP), 0.765 (TN), and 0.709 (OM), respectively (Supplementary Table 2).

The heatmap based on Spearman's analysis was used to analyze the relationship between soil physiochemistry properties and the top 20 bacterial genera of rhizosphere soil and bulk soil (Figure 6B). Group I including *Flavobacterium, Devosia, Hydrogenophaga, Ensifer, Sphingomonas, Streptomyces, Bacillus, Dongia, Pseudarthrobacter, Methylotenera*, and *Lechevalieria* presented the negative correlation of pH and the positive correlation of OM. AP, TN, and AK did not affect the relative abundance of bacteria in group I, except *Sphingomonas* and *Streptomyces*. Compared with group I, group II, including *Polycyclovorans, Haliangium, Bryobacter, Pseudomonas, MND1*, and *Nitrospira*, exhibited the opposite trend, and their relative abundance was positively correlated with pH and was negatively correlated with OM. Besides, AP, TN, and AK had no impact on the relative abundance of bacteria in group II, except *Polycyclovorans* and *Haliangium*.

## Discussion

Continuous cropping significantly inhibited the growth of stevia and the yield of SVglys; and soil deterioration plays an unquestioned role (Tan et al., 2021). Except pH, the OM, TN, AP, and AK of Y8 were all significantly lower than that of Y2 in the rhizosphere soil, and similar trends were observed in bulk soil except for OM. Previous studies have shown that soil pH exerted a strong influence on the structure of soil microbial communities, and it correlates with the diversity and richness of soil bacterial communities found in different ecosystems (Fierer and Jackson, 2006; Lauber et al., 2009; Tripathi et al., 2012). Future experiments with more continuous cropping years of stevia might help in clarifying the potential of soil pH to regulate the soil bacterial community.

In this study, the total number of OTUs found in the stevia root was significantly lower than in the soil, and the OTUs shared by root, rhizosphere soil, and bulk soil were close in both Y2 and Y8. It suggested that continuous cropping did not affect the stability distribution of bacteria between root and soil, which might be explained by the structural stability of bacterial communities in different niches of plants (Beckers et al., 2017). Moreover, a significantly higher difference value of the OTU number was found in the rhizosphere soil than in the root and the bulk soil, suggesting that a broad diversity of bacterial activities is enhanced in strict contact with plant roots (Li et al., 2014). The small differences in the number of unique OTUs of roots and bulk soils between Y2 and Y8 can be ascribed to the fact that the rhizosphere soil exhibits intense biological and chemical activities (Prashar et al., 2014), which is consistent with the soil physicochemical properties.

Root endophytes are an important part of the plant root microecosystem, and the root microbiome is predominantly assembled from the external microbes in the soil (Edwards et al., 2015). Obviously, the roots and soil bacterial communities were clearly separated from each other, indicating a significant impact of stevia roots on selecting and shaping the rhizosphere bacterial community. Previous study indicated that the diversity of endophytic bacteria in roots of *Panax notoginseng* was significantly lower than in rhizosphere soil and had less change in the planting time (Tan et al., 2017), which was similar to the results of our study. Moreover, soil environmental factors are the main driving forces for the change in microbial communities (Jiao et al., 2016). Continuous cropping affects the diversity rather than the richness of soil bacteria, which might be due to the selectivity of soil microorganisms in the soil environment. The specific environment became more suitable for the survival of specific bacteria, such as *Bacteroidetes* and *Firmicutes*, which were enriched in nutrient-rich soil and poorly structured soil, respectively (Marasco et al., 2013; Griffith et al., 2017). In the previous study, the diversity of *Nitrospirae* and *Elusimicrobia* in rhizosphere soil of diseased plants was higher after continuous cropping of *Atractylodes macrocephala* (Yuan et al., 2019); *Planctomycete*, one soil oligotroph (Fierer et al., 2007), was found in environments with pH values ranging from below 3 to above 11 (Bohorquez et al., 2012), which was difficult for the plant to grow. Coincidently, *Nitrospirae, Elusimicrobia*, and *Planctomycete* of rhizosphere soil significantly increased after Y8, which was also consistent with the decrease in soil nutrient content after Y8.

At the genus level, continuous cropping also changed the relative abundance of predominant bacteria. In the previous studies, *Streptomyces* (*Actinobacteria*) is the most important bacterial community in soil and has great potential for bioremediation of toxic compounds (Álvarez et al., 2012); *Flavobacterium* (*Bacteroidetes*), a kind of plant growth-promoting bacteria (PGPB), exhibits significant biocontrol activity and affects many events occurring during the plant life cycle (Jeong et al., 2019; Kim et al., 2020); *Sphingomonas* (*Bacteroidetes*) resisted plant diseases and promoted plant growth (Khan et al., 2014; Zhou et al., 2016); and *Devosia* is well known for its dominance in soil habitats contaminated with various toxins and is best characterized for its bioremediation potential (Talwar et al., 2020). Unfortunately, our study showed that the above four genera were not abundant in Y8. The desolating microbial populations decrease soil productivity and crop yields over time. In addition, *Polycyclovorans* (*Proteobacteria*) may be the obligate hydrocarbonoclastic bacteria and exhibit a narrow nutritional spectrum (Thompson et al., 2018), which is considered to be the bacterial host related to soil pollution (Costeira et al., 2019); *Haliangium* is sensitive to antibiotic disturbance (Uddin et al., 2019), and it produced antifungal substances that act against phytopathogens (Ma et al., 2018); *Methylobacillus* was involved in polycyclic aromatic hydrocarbon (PAH) degradation and might have the ability to restore the contaminated soil (Lu et al., 2019). In this study, the relative abundance of *Polycyclovorans, Haliangium*, and *Methylobacillus* significantly increased in rhizosphere soil after 8 years of continuous stevia cropping. It suggested that the adverse effects caused by long-term continuous cropping might be mitigated by the increase of the above degrading bacteria. Notably, as a kind of beneficial bacteria, *Nitrospira* is significantly influenced by soil pH, exhibiting the strongest biological activity under pH neutral or slightly alkaline conditions, and its abundance is easily inhibited by a high nutritional environment (Simonin et al., 2015; Bai et al., 2020; Yin et al., 2021). Soil with the highest pH and lower nutrient content after 8 years of continuous stevia cropping may explain the significant increase of *Nitrospira* in soil.

Soil ecosystems are formed by complex interactions between biological communities and physicochemical variables, which jointly determine the overall quality of soil (Hermans et al., 2020). The complex regulatory network of the soil bacterial community is mainly related to soil fertility and ecological function, especially soil pH (Kim et al., 2016; Ren et al., 2018). We concluded that the soil bacterial community was significantly affected by soil physicochemical properties including pH, AK, and AP, which was consistent with previous studies (Rousk et al., 2010; Shen et al., 2013; Li et al., 2019). It also indicated that continuous cropping may interfere with the composition and diversity of the microbial community by affecting soil pH. As a result, continuous cropping played a key role in the composition, chemical properties, and utilization efficiency of the matrix and the imbalance of bacterial community function, and it further exhibited an adverse influence on the growth of stevia and the content of SVglys.

In summary, the long-term continuous stevia cropping changed the bacterial communities in the root and soil, enriched the harmful bacteria, and reduced the beneficial bacteria. Therefore, measures must be taken to restore soil microbial communities (Shen et al., 2021). Results of this study may be used to explore reasonable management measures, such as rational fertilization, microbial fertilizer application, and rotation or intercropping for eventual relief of the long-term monoculture problems of stevia plants. In addition, the stevia rhizosphere exhibited strong impacts on the population of bacteria and fungi (Deng et al., 2018); both bacterial and fungal communities were shaped by the same edaphic factors (Zheng et al., 2019). Thus, the whole microbial communities including bacteria, fungi, and archaea shall be studied in the future, together with the dynamics of plant developmental stages.

## Conclusion

This study analyzed the variation of rhizosphere bacterial community structure of stevia under Y2 and Y8. The relationship of the bacterial community, soil physicochemical properties, and SVgly content was investigated. Our study indicated that during the long-term continuous cropping, bacterial communities shaped into great compositional variations in the stevia root, rhizosphere soil, and bulk soil. The soil physicochemical properties were also greatly transformed with continuous cropping, and it affected the yield and content of SVglys. Our study provides a theoretical reference for the alleviation of continuous cropping obstacles in stevia.

## Data Availability Statement

The datasets presented in this study can be found in online repositories. The names of the repository/repositories and accession number(s) can be found below: https://www.ncbi.nlm.nih.gov/, PRJNA824437.

## Author Contributions

XX: validation and visualization. XX and QL: investigation. XX, SJ, and ZH: data curation. QL and XH: project administration. QL, ZH, and XH: funding acquisition. XX, XH, MF, ZX, CY, and ZZ: manuscript writing and editing. All authors contributed to the article and approved the submitted version.

## Funding

This study was supported by the project Environmental assessment of *Stevia rebaudiana* production in China (20190012), which was delivered in collaboration with Earthwatch Europe, and the Postgraduate Education Reform and Quality Improvement Project of Henan Province (Yu degree [2018] No. 23). This study was also supported by the Shennongjia Academy of Forestry, Hubei, China (No. SAF202102 to XH) and the Hubei Technology Innovation Center for Agricultural Sciences−2020 key technology research and demonstration project of safe and efficient production of genuine medicinal materials (No. 2020-620-000-002-04 to XH).

## Conflict of Interest

The authors declare that the research was conducted in the absence of any commercial or financial relationships that could be construed as a potential conflict of interest.

## Publisher's Note

All claims expressed in this article are solely those of the authors and do not necessarily represent those of their affiliated organizations, or those of the publisher, the editors and the reviewers. Any product that may be evaluated in this article, or claim that may be made by its manufacturer, is not guaranteed or endorsed by the publisher.

## References

[B1] Álvarez A. Yañez M. L. Benimeli C. S. Amoroso M.J. (2012). Maize plants (Zea mays) root exudates enhance lindane removal by native Streptomyces strains. Int. Biodeterior. Biodegrad. 66, 14–18. 10.1016/j.ibiod.2011.10.001

[B2] Bai Y. C. Chang Y. Y. Hussain M. Lu B. Zhang J. P. Song X. B.. (2020). Soil chemical and microbiological properties are changed by long-term chemical fertilizers that limit ecosystem functioning. Microorganisms 8, 694. 10.3390/microorganisms8050694 32397341 PMC7285516

[B3] Beckers B. Op De Beeck M. Weyens N. Boerjan W. Vangronsveld J. (2017). Structural variability and niche differentiation in the rhizosphere and endosphere bacterial microbiome of field-grown poplar trees. Microbiome 5, 25. 10.1186/s40168-017-0241-2 28231859 PMC5324219

[B4] Berendsen R. L. Pieterse C. M. Bakker P. A. (2012). The rhizosphere microbiome and plant health. Trends Plant Sci. 17, 478–486. 10.1016/j.tplants.2012.04.001 22564542

[B5] Bohorquez L. C. Delgado-Serrano L. Lopez G. Osorio-Forero C. Klepac-Ceraj V. Kolter R.. (2012). In-depth characterization via complementing culture-independent approaches of the microbial community in an acidic hot spring of the Colombian Andes. Microb. Ecol. 63, 103–115. 10.1007/s00248-011-9943-3 21947461

[B6] Bolyen E. Rideout J. R. Dillon M. R. Bokulich N. A. Abnet C. C. Al-Ghalith G. A.. (2019). Reproducible, interactive, scalable and extensible microbiome data science using QIIME 2. Nat. Biotechnol. 37, 852–857. 10.1038/s41587-019-0209-9 31341288 PMC7015180

[B7] Castrillo G. Teixeira P. J. Paredes S. H. Law T. F. De Lorenzo L. Feltcher M. E.. (2017). Root microbiota drive direct integration of phosphate stress and immunity. Nature 543, 513–518. 10.1038/nature21417 28297714 PMC5364063

[B8] Costeira R. Doherty R. Allen C. C. R. Larkin M. J. Kulakov L. A. (2019). Analysis of viral and bacterial communities in groundwater associated with contaminated land. Sci. Total Environ. 656, 1413–1426. 10.1016/j.scitotenv.2018.11.429 30625669

[B9] Deng S. Ke T. Li L. Cai S. Zhou Y. Liu Y.. (2018). Impacts of environmental factors on the whole microbial communities in the rhizosphere of a metal-tolerant plant: *Elsholtzia haichowensis* Sun. Environ. Pollut. 237, 1088–1097. 10.1016/j.envpol.2017.11.037 29153474

[B10] Díaz-Gutiérrez C. Hurtado A. Ortíz A. Poschenrieder C. Arroyave C. Peláez C. (2020). Increase in steviol glycosides production from *Stevia rebaudiana* Bertoni under organo-mineral fertilization. Ind. Crop. Prod. 147, 112220. 10.1016/j.indcrop.2020.112220

[B11] Dong L. Xu J. Feng G. Li X. Chen S. (2016). Soil bacterial and fungal community dynamics in relation to Panax notoginseng death rate in a continuous cropping system. Sci Rep. 6, 31802. 10.1038/srep31802 27549984 PMC4994099

[B12] Edgar R. C. (2010). Search and clustering orders of magnitude faster than BLAST. Bioinformatics 26, 2460–2461. 10.1093/bioinformatics/btq461 20709691

[B13] Edwards J. Johnson C. Santos-Medellin C. Lurie E. Podishetty N. K. Bhatnagar S.. (2015). Structure, variation, and assembly of the root-associated microbiomes of rice. Proc. Natl. Acad. Sci. U. S. A. 112, E911–920. 10.1073/pnas.1414592112 25605935 PMC4345613

[B14] Fan S. Zhao F. Zhang J. Shang W. Hu X. (2021). American ginseng root rot caused by *Fusarium redolens* in China. Plant Dis. 105, 2734. 10.1094/PDIS-12-20-2600-PDN33441010

[B15] Fierer N. Bradford M. A. Jackson R. B. (2007). Toward an ecological classification of soil bacteria. Ecology 88, 1354–1364. 10.1890/05-1839 17601128

[B16] Fierer N. Jackson R. B. (2006). The diversity and biogeography of soil bacterial communities. Proc. Natl. Acad. Sci. U. S. A. 103, 626–631. 10.1073/pnas.0507535103 16407148 PMC1334650

[B17] Garcia A. Polonio J. C. Polli A. D. Santos C. M. Rhoden S. A. Quecine M. C.. (2016). Rhizosphere bacteriome of the medicinal plant *Sapindus saponaria* L. revealed by pyrosequencing. Genet. Mol. Res. 15, 1–9. 10.4238/gmr15049020 27819730

[B18] Griffith J. C. Lee W. G. Orlovich D. A. Summerfield T. C. (2017). Contrasting bacterial communities in two indigenous Chionochloa (Poaceae) grassland soils in New Zealand. PLoS ONE 12, e0179652. 10.1371/journal.pone.0179652 28658306 PMC5489180

[B19] Hermans S. M. Buckley H. L. Case B. S. Curran-Cournane F. Taylor M. Lear G. (2020). Using soil bacterial communities to predict physico-chemical variables and soil quality. Microbiome 8, 79. 10.1186/s40168-020-00858-1 32487269 PMC7268603

[B20] Jeong J. J. Sajidah S. Oh J. Y. Sang M. K. Kim K. S. Kim K. D. (2019). Complete genome sequence data of Flavobacterium anhuiense strain GSE09, a volatile-producing biocontrol bacterium isolated from cucumber (*Cucumis sativus*) root. Data Brief. 25, 104270. 10.1016/j.dib.2019.104270 31388522 PMC6676235

[B21] Jiao S. Liu Z. Lin Y. Yang J. Chen W. Wei G. (2016). Bacterial communities in oil contaminated soils: Biogeography and co-occurrence patterns. Soil Biol. Biochem. 98, 64–73. 10.1016/j.soilbio.2016.04.005

[B22] Karimi M. Ahmadi A. Hashemi J. Abbasi A. Tavarini S. Pompeiano A.. (2019). Plant growth retardants (PGRs) affect growth and secondary metabolite biosynthesis in *Stevia rebaudiana* Bertoni under drought stress. S. Afr. J. Bot. 121, 394–401. 10.1016/j.sajb.2018.11.028

[B23] Khan A. L. Waqas M. Kang S. M. Al-Harrasi A. Hussain J. Al-Rawahi A.. (2014). Bacterial endophyte Sphingomonas sp. LK11 produces gibberellins and IAA and promotes tomato plant growth. J. Microbiol. 52, 689–695. 10.1007/s12275-014-4002-7 24994010

[B24] Kim J.-E. Woo O.-G. Bae Y. Keum H. L. Chung S. Sul W. J.. (2020). Enhanced drought and salt stress tolerance in arabidopsis by *Flavobacterium crocinum* HYN0056T. J. Plant Biol. 63, 63–71. 10.1007/s12374-020-09236-8

[B25] Kim J. M. Roh A. S. Choi S. C. Kim E. J. Choi M. T. Ahn B. K.. (2016). Soil pH and electrical conductivity are key edaphic factors shaping bacterial communities of greenhouse soils in Korea. J. Microbiol. 54, 838–845. 10.1007/s12275-016-6526-5 27888456

[B26] Lauber C. Hamady M. Knight R. Fierer N. (2009). Pyrosequencing-based assessment of soil pH as a predictor of soil bacterial community structure at the continental scale. Appl. Environ. Microbiol. 75, 5111–5120. 10.1128/AEM.00335-09 19502440 PMC2725504

[B27] Li C. Chen G. Zhang J. Zhu P. Bai X. Hou Y.. (2021). The comprehensive changes in soil properties are continuous cropping obstacles associated with American ginseng (*Panax quinquefolius*) cultivation. Sci Rep. 11, 5068. 10.1038/s41598-021-84436-x 33658624 PMC7930255

[B28] Li M. Wang Q. Liu Z. Pan X. Zhang Y. (2019). Silicon application and related changes in soil bacterial community dynamics reduced ginseng black spot incidence in Panax ginseng in a short-term study. BMC Microbiol. 19, 263. 10.1186/s12866-019-1627-z 31771526 PMC6880445

[B29] Li X. Rui J. Xiong J. Li J. He Z. Zhou J.. (2014). Functional potential of soil microbial communities in the maize rhizosphere. PLoS ONE 9, e112609. 10.1371/journal.pone.0112609 25383887 PMC4226563

[B30] Liu J. Li J.-W. Tang J. (2010). Ultrasonically assisted extraction of total carbohydrates from Stevia rebaudiana Bertoni and identification of extracts. Food Bioprod. Process 88, 215–221. 10.1016/j.fbp.2009.12.005

[B31] Liu N. Shao C. Sun H. Liu Z. Guan Y. Wu L.. (2020). Arbuscular mycorrhizal fungi biofertilizer improves American ginseng (*Panax quinquefolius* L.) growth under the continuous cropping regime. Geoderma 363, 114155. 10.1016/j.geoderma.2019.114155

[B32] Lu C. Hong Y. Liu J. Gao Y. Ma Z. Yang B.. (2019). A PAH-degrading bacterial community enriched with contaminated agricultural soil and its utility for microbial bioremediation. Environ. Pollut. 251, 773–782. 10.1016/j.envpol.2019.05.044 31121542

[B33] Ma M. Jiang X. Wang Q. Guan D. Li L. Ongena M.. (2018). Isolation and identification of PGPR strain and its effect on soybean growth and soil bacterial community composition. Int. J. Agric. Biol. 20, 1289–1297. 10.17957/IJAB/15.0627

[B34] Marasco R. Rolli E. Fusi M. Cherif A. Abou-Hadid A. El-Bahairy U.. (2013). Plant growth promotion potential is equally represented in diverse grapevine root-associated bacterial communities from different biopedoclimatic environments. Biomed Res. Int. 2013, 491091. 10.1155/2013/491091 23878810 PMC3708380

[B35] Prashar P. Kapoor N. Sachdeva S. (2014). Rhizosphere: its structure, bacterial diversity and significance. Rev. Environ. Sci. Biotechnol. 13, 63–77. 10.1007/s11157-013-9317-z19185328

[B36] Qiao Q. Wang F. Zhang J. Chen Y. Zhang C. Liu G.. (2017). The variation in the rhizosphere microbiome of cotton with soil type, genotype and developmental stage. Sci Rep. 7, 3940. 10.1038/s41598-017-04213-7 28638057 PMC5479781

[B37] Quast C. Pruesse E. Yilmaz P. Gerken J. Schweer T. Yarza P.. (2013). The SILVA ribosomal RNA gene database project: improved data processing and web-based tools. Nucleic Acids Res. 41, D590–D596. 10.1093/nar/gks1219 23193283 PMC3531112

[B38] Ren B. Hu Y. Chen B. Zhang Y. Thiele J. Shi R.. (2018). Soil pH and plant diversity shape soil bacterial community structure in the active layer across the latitudinal gradients in continuous permafrost region of Northeastern China. Sci Rep. 8, 5619. 10.1038/s41598-018-24040-8 29618759 PMC5884794

[B39] Rousk J. Baath E. Brookes P. C. Lauber C. L. Lozupone C. Caporaso J. G.. (2010). Soil bacterial and fungal communities across a pH gradient in an arable soil. ISME J. 4, 1340–1351. 10.1038/ismej.2010.58 20445636

[B40] Shen C. Xiong J. Zhang H. Feng Y. Lin X. Li X.. (2013). Soil pH drives the spatial distribution of bacterial communities along elevation on Changbai Mountain. Soil Biol. Biochem. 57, 204–211. 10.1016/j.soilbio.2012.07.013

[B41] Shen W. Hu M. Qian D. Xue H. Gao N. Lin X. (2021). Microbial deterioration and restoration in greenhouse-based intensive vegetable production systems. Plant Soil 463, 1–18. 10.1007/s11104-021-04933-w

[B42] Shen Z. Ruan Y. Xue C. Zhong S. Li R. Shen Q. (2015). Soils naturally suppressive to banana Fusarium wilt disease harbor unique bacterial communities. Plant Soil 393, 21–33. 10.1007/s11104-015-2474-9

[B43] Simonin M. Le Roux X. Poly F. Lerondelle C. Hungate B. A. Nunan N.. (2015). Coupling between and among ammonia oxidizers and nitrite oxidizers in grassland mesocosms submitted to elevated CO2 and nitrogen supply. Microb. Ecol. 70, 809–818. 10.1007/s00248-015-0604-9 25877793

[B44] Talwar C. Nagar S. Kumar R. Scaria J. Lal R. Negi R. K. (2020). Defining the environmental adaptations of genus devosia: insights into its expansive short peptide transport system and positively selected genes. Sci. Rep. 10, 1151. 10.1038/s41598-020-58163-8 31980727 PMC6981132

[B45] Tan G. Liu Y. Peng S. Yin H. Meng D. Tao J.. (2021). Soil potentials to resist continuous cropping obstacle: three field cases. Environ. Res. 200, 111319. 10.1016/j.envres.2021.111319 34052246

[B46] Tan Y. Cui Y. Li H. Kuang A. Li X. Wei Y.. (2017). Diversity and composition of rhizospheric soil and root endogenous bacteria in *Panax notoginseng* during continuous cropping practices. J. Basic Microbiol. 57, 337–344. 10.1002/jobm.201600464 28060404

[B47] Tavarini S. Sgherri C. Ranieri A. M. Angelini L. G. (2015). Effect of nitrogen fertilization and harvest time on steviol glycosides, flavonoid composition, and antioxidant properties in stevia rebaudiana bertoni. J. Agric. Food Chem. 63, 7041–7050. 10.1021/acs.jafc.5b02147 26194177

[B48] Thompson H. F. Lesaulnier C. Pelikan C. Gutierrez T. (2018). Visualisation of the obligate hydrocarbonoclastic bacteria *Polycyclovorans algicola* and *Algiphilus aromaticivorans* in co-cultures with micro-algae by CARD-FISH. J. Microbiol. Methods 152, 73–79. 10.1016/j.mimet.2018.07.016 30063956

[B49] Tripathi B. M. Kim M. Singh D. Lee-Cruz L. Lai-Hoe A. Ainuddin A. N.. (2012). Tropical soil bacterial communities in Malaysia: pH dominates in the equatorial tropics too. Microb. Ecol. 64, 474–484. 10.1007/s00248-012-0028-8 22395784

[B50] Uddin M. Chen J. Qiao X. Tian R. Arafat Y. Yang X. (2019). Bacterial community variations in paddy soils induced by application of veterinary antibiotics in plant-soil systems. Ecotox. Environ. Safe 167, 44–53. 10.1016/j.ecoenv.2018.09.101 30292975

[B51] Wacal C. Ogata N. Sasagawa D. Handa T. Basalirwa D. Acidri R.. (2019). Seed yield, crude protein and mineral nutrient contents of sesame during a two-year continuous cropping on upland field converted from a paddy. Field Crop. Res. 240, 125–133. 10.1016/j.fcr.2019.06.004

[B52] Wang R. Xiao Y. Lv F. Hu L. Wei L. Yuan Z.. (2018). Bacterial community structure and functional potential of rhizosphere soils as influenced by nitrogen addition and bacterial wilt disease under continuous sesame cropping. Appl. Soil Ecol. 125, 117–127. 10.1016/j.apsoil.2017.12.014

[B53] Wang S. Cheng J. Li T. Liao Y. (2020). Response of soil fungal communities to continuous cropping of flue-cured tobacco. Sci. Rep. 10, 19911. 10.1038/s41598-020-77044-8 33199813 PMC7669846

[B54] Wang Z. Zhu Y. Jing R. Wu X. Li N. Liu H.. (2021). High-throughput sequencing-based analysis of the composition and diversity of endophytic bacterial community in seeds of upland rice. Arch. Microbiol. 203, 609–620. 10.1007/s00203-020-02058-9 32995980

[B55] Wu H. Qin X. Wang J. Wu L. Chen J. Fan J.. (2019). Rhizosphere responses to environmental conditions in Radix pseudostellariae under continuous monoculture regimes. Agric. Ecosyst. Environ. 270–271, 19–31. 10.1016/j.agee.2018.10.014

[B56] Wu Z. Hao Z. Zeng Y. Guo L. Huang L. Chen B. (2015). Molecular characterization of microbial communities in the rhizosphere soils and roots of diseased and healthy *Panax notoginseng*. Antonie Van Leeuwenhoek 108, 1059–1074. 10.1007/s10482-015-0560-x 26296378

[B57] Xia Y. He X. Feng Z. Zhang Q. Yang H. (2019). A comprehensive analysis of the microbial diversity in natural and engineered ecosystems based on high-throughput sequencing of 16S rRNA gene. Int. Biodeterior. Biodegrad. 140, 160–168. 10.1016/j.ibiod.2019.03.018

[B58] Xiong W. Zhao Q. Zhao J. Xun W. Li R. Zhang R.. (2015). Different continuous cropping spans significantly affect microbial community membership and structure in a vanilla-grown soil as revealed by deep pyrosequencing. Microb. Ecol. 70, 209–218. 10.1007/s00248-014-0516-0 25391237

[B59] Yang Y. Chen X. Chen J. Xu H. Li J. Zhang Z. (2011). Differential miRNA expression in *Rehmannia glutinosa* plants subjected to continuous cropping. BMC Plant Biol. 11, 53. 10.1186/1471-2229-11-53 21439075 PMC3078876

[B60] Yin C. Schlatter D. C. Kroese D. R. Paulitz T. C. Hagerty C. H. (2021). Impacts of lime application on soil bacterial microbiome in dryland wheat soil in the Pacific Northwest. Appl. Soil Ecol. 168, 104113. 10.1016/j.apsoil.2021.104113

[B61] Yuan X. F. Song T. J. Yang J. S. Huang X. G. Shi J. Y. (2019). Changes of microbial community in the rhizosphere soil of *Atractylodes macrocephala* when encountering replant disease. S. Afr. J. Bot. 127, 129–135. 10.1016/j.sajb.2019.08.046

[B62] Zheng Q. Hu Y. Zhang S. Noll L. Böckle T. Dietrich M.. (2019). Soil multifunctionality is affected by the soil environment and by microbial community composition and diversity. Soil Biol. Biochem. 136, 107521. 10.1016/j.soilbio.2019.107521 31700196 PMC6837881

[B63] Zhou L. Li H. Zhang Y. Han S. Xu H. (2016). Sphingomonas from petroleum-contaminated soils in Shenfu, China and their PAHs degradation abilities. Braz. J. Microbiol. 47, 271–278. 10.1016/j.bjm.2016.01.001 26991271 PMC4874584

[B64] Zhu L. Yan H. Zhou G. S. Jiang C. H. Liu P. Yu G.. (2021). Insights into the mechanism of the effects of rhizosphere microorganisms on the quality of authentic Angelica sinensis under different soil microenvironments. BMC Plant Biol. 21, 285. 10.1186/s12870-021-03047-w 34157988 PMC8220839

