# Supplementary material for: The Deterioration of Agronomical Traits of the Continuous Cropping of Stevia Is Associated With the Dynamics of Soil Bacterial Community
Source: Front Microbiol. 2022 Jun 16;13:917000. doi: 10.3389/fmicb.2022.917000 (PMC9277660; doi:10.3389/fmicb.2022.917000)
Supplement: Supplementary file 1 [file Data_Sheet_1.docx]

**SUPPLEMENTARY TABLE 1** | The statistical comparison of microbiome compositions among all the six groups in six taxonomy classification levels.

| group | kingdom | phylum | class | order | family | genus |
| --- | --- | --- | --- | --- | --- | --- |
| Y2.R | 673.67±100.31 d | 634.67±95.78 d | 632.33±95.81 d | 610.17±91.14 d | 566.50±77.62 d | 494.33±64.58 d |
| Y8.R | 789.50±270.02 d | 741.17±271.81 d | 738.33±272.28 d | 714.50±270.43 d | 678.67±266.60 d | 607.00±250.80 d |
| Y2.RS | 22350.50±2703.35 a | 22253.00±2722.75 a | 22140.83±2724.73 a | 20900.00±2703.40 a | 19263.67±2557.89 a | 15853.67±2096.23 a |
| Y8.RS | 19193.67±2464.56 b | 19081.83±2457.63 b | 18962.33±2438.69 b | 17465.83±2358.56 b | 15835.17±2352.59 b | 12523.83±2062.25 b |
| Y2.BS | 17137.33±2034.45 bc | 17035.00±2030.82 bc | 16856.33±1993.09 bc | 14964.33±1704.52 c | 12930.50±1546.68 c | 9479.17±1102.55 c |
| Y8.BS | 16558.67±2595.21 c | 16444.00±2593.21 c | 16217.33±2569.91 c | 14246.17±2464.93 c | 12248.33±2322.55 c | 8946.50±1839.84 c |

*Note: Y2.R, root of 2 years continuous cropping; Y8.R, root of 8 years continuous cropping; Y2.RS, rhizosphere soil of 2 years continuous cropping; Y8.RS, rhizosphere soil of 8 years continuous cropping; Y2.BS, bulk soil of 2 years continuous cropping; Y8.BS, bulk soil of 8 years continuous cropping. The different lowercase letters in the same column indicate significant differences between different treats (p < 0.05).*

**SUPPLEMENTARY TABLE 2** | Relationships between soil bacteria and environmental factors as revealed by Mantel.

| Factor | r^2^ | *P* value |
| --- | --- | --- |
| pH | 0.929 | 0.001 |
| OM | 0.709 | 0.001 |
| TN | 0.765 | 0.001 |
| AP | 0.881 | 0.001 |
| AK | 0.922 | 0.001 |

*Note: pH, soil pH; OM, organic matter; TN, total nitrogen; AP, soil available phosphors; AK, soil available potassium.*
